# Skeletal muscle metastasis as a first site of recurrence of cervical cancer: A case report and review of the literature

**DOI:** 10.1097/MD.0000000000020056

**Published:** 2020-05-08

**Authors:** Nagisa Omokawa, Seiji Mabuchi, Kana Iwai, Naoki Kawahara, Ryuji Kawaguchi, Sumire Sugimoto, Chiho Ohbayashi, Kanya Honoki, Akira Nagai, Hiroshi Kobayashi

**Affiliations:** a Department of Obstetrics and Gynecology; b Department of Diagnostic Pathology; c Department of Orthopedics, Nara Medical University, Kashihara city, Nara; d Department of Obstetrics and Gynecology, Osaka Habikino Medical Center, Osaka, Japan.

**Keywords:** cervical cancer, metastasis, oblique muscle, treatment

## Abstract

**Rationale:**

Cervical cancer primarily spreads through direct invasion or via local lymphatics, and hematogenous metastasis is infrequent. Previous reports have shown that lung, liver, and bone are the organs most frequently affected by hematogenous metastasis of cervical cancer, while skeletal muscle is very rarely involved.

**Patient concerns:**

A 75-year-old Japanese woman presented with a painful muscular mass in her right lower abdomen. Five years ago, she was treated for her International Federation of Gynecology and Obstetrics stage IB2 cervical adenocarcinoma with radical surgery plus adjuvant chemotherapy.

**Diagnoses:**

The patient was diagnosed with isolated oblique muscle metastasis from cervical adenocarcinoma as a first site of recurrence.

**Interventions:**

The patient was treated with salvage surgery consisting of partial resection of the oblique muscle and ilium. The tumor was completely excised with an adequate surgical margin by a partial resection of the oblique muscle and ilium

**Outcomes:**

The patient is currently free of disease at 10 months after the development of recurrent disease.

**Lessons:**

We describe a rare case of isolated oblique muscle metastasis as a first site of recurrence of the International Federation of Gynecology and Obstetrics stage IB2 cervical adenocarcinoma, which was successfully treated with surgery. Although skeletal muscle metastasis is rare, this condition should be considered during the follow-up period, especially when patients complain of muscular pain with insidious progression. The present case and our literature review highlighted the possibility that loco-regional treatment may be curative for selected recurrent cervical cancer developed in skeletal muscles.

## 1 Introduction

Cervical cancer primarily spreads by direct invasion or through the local lymphatics, and hematogenous metastasis is uncommon, with lung, liver, and bone being the most frequent sites of hematogenous metastasis.^[1]^ Although the skeletal muscles represent up to 50% of the total body weight and receive a large percentage of total cardiac output, skeletal muscle metastasis is extremely rare in patients with solid malignancies, including cervical cancer, with a reported incidence of less than 1%.^[2]^ Patients who present with untreated cervical cancer and skeletal muscle metastasis are diagnosed as the International Federation of Gynecology and Obstetrics (FIGO) stage IVB, and they usually also have other metastatic lesions and a dismal prognosis.^[2,3]^ However, in the setting of recurrent disease, it seems that skeletal muscle metastasis does not always indicate systemic disease (Table 1), and successful results of loco-regional treatment have been reported in patients with isolated skeletal muscle metastasis.^[4–21]^

**Table 1:**
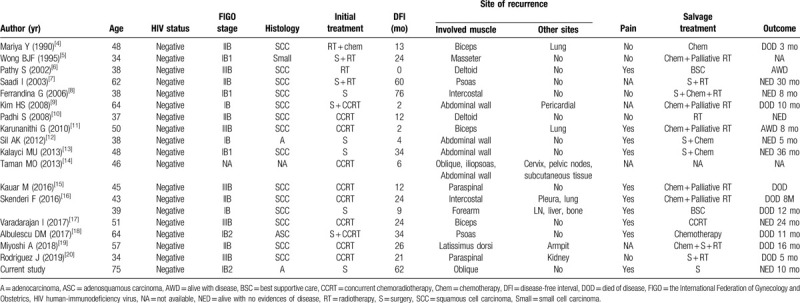
Summary of reported cases with skeletal muscle metastasis as sites of recurrence of uterine cervical cancer.

In this report, we describe our experience with isolated oblique muscle metastasis as a first site of recurrence of FIGO stage IB2 cervical adenocarcinoma, which was successfully treated by surgery. Moreover, based on a literature review, we also provide information about the management of skeletal muscle metastasis in cervical cancer patients in a recurrent setting.

### 1.1 Case report

**Figure 1:**
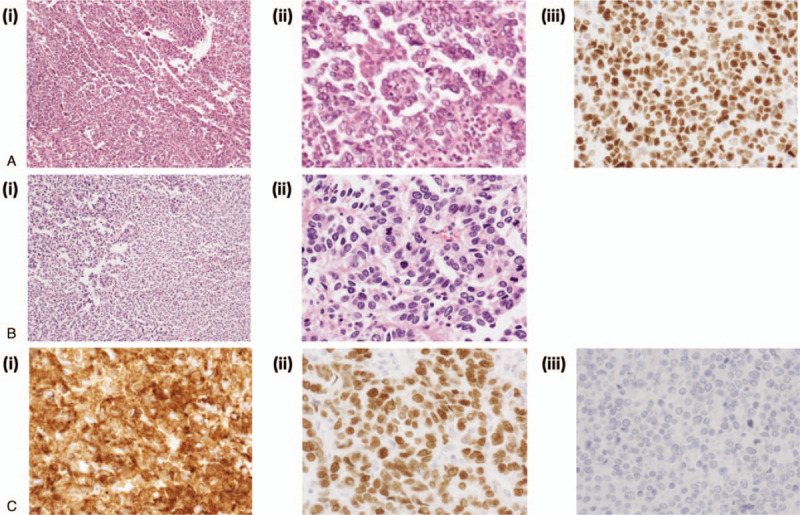
A, (i-ii) Hematoxylin and eosin stained section of the primary tumor. The tumor has high-grade nuclear atypia and a complex papillary architecture with cellular budding [(i), x10; (ii), x40]. (iii) Immunohistochemical analysis revealed that the tumor cells were diffusely positive for p53 (x 40), suggesting adenocarcinoma of the uterine cervix. B, Hematoxylin and eosin stained section of the resected oblique muscle tumor, featuring infiltration of cells with pleomorphic hyperchromatic nuclei. There is solid proliferation of neoplastic cells with focal papillary or glandular structures, consistent with a metastatic disease from cervical adenocarcinoma [(i), x10; (ii), x40]. C, immunohistochemical findings of the resected oblique muscle tumor (x 40). Tumor cells were diffusely positive for cytokeratin AE1/AE3 (i) and p53 (ii), but negative for estrogen receptor (iii), showing the similar immunoreactivity profile as the primary cervical tumor.

A 70-year-old Japanese woman presented with postmenopausal vaginal bleeding. She had a history of diabetes mellitus and hepatitis C. On evaluation, she was found to have a 6 cm friable cervical mass. There was no parametrial involvement and rectal examination was normal. Pelvic magnetic resonance imaging (MRI) showed a 6 cm cervical mass limited to the uterine cervix and an enlarged pelvic lymph node (left obturator node). Biopsies of the lesion demonstrated adenocarcinoma. Computed tomography (CT) scans of the abdomen and chest showed no evidence of metastatic disease, and laboratory findings were unremarkable. A diagnosis of FIGO stage IB2 cervical cancer was made from these findings, and she underwent radical hysterectomy with bilateral salpingo-oophorectomy. Pathological examination of the surgical specimen revealed invasive serous carcinoma of the uterine cervix with right obturator lymph node metastasis (pT1b2N1M0) (Fig. 1). Deep stromal invasion and lymphovascular space involvement were also noted, but the surgical margin, fallopian tubes, and both ovaries were free of tumor. Because of the pathological risk factors, adjuvant therapy was recommended, and she received adjuvant chemotherapy with 6 cycles of carboplatin (AUC5) plus paclitaxel (175 mg/kg).

**Figure 2:**
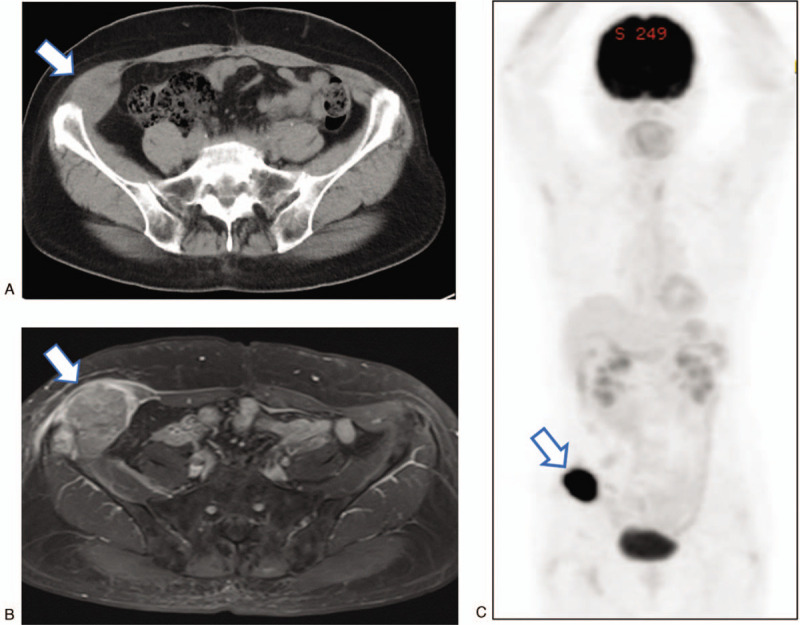
Images of the metastasis in the right oblique muscle. A, Contrast-enhanced computed tomography. B, Contrast-enhanced magnetic resonance imaging (T1-weighted). Arrows indicate a 5 x 6 cm enhancing tumor with direct involvement of the ilium. C, 2-deoxy-2-[^18^F] fluoro-D-glucose position emission tomography shows a hypermetabolic lesion in the right oblique muscle. Arrows indicate an oblique muscle tumor.

**Figure 3:**
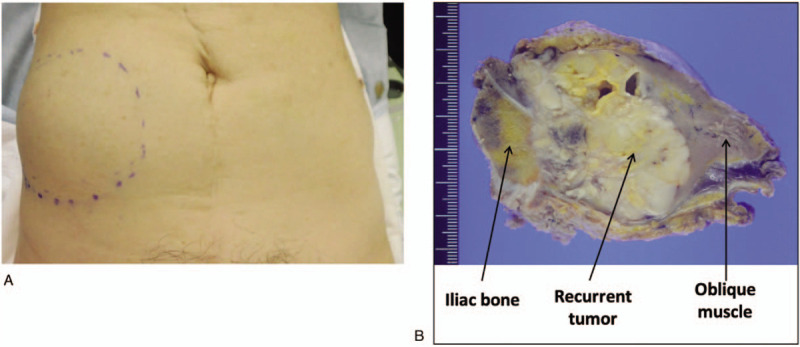
A, Appearance of the recurrent tumor in the right iliac region. B and C, Oblique muscle tumor with direct involvement of the ilium (B, intraoperative view: C, resected tumor).

Sixty-two months after the initial operation, she presented with a painful mass in her right lower abdomen. On examination, she had a 5 cm nodular lesion in the right anterior abdominal wall close to the ilium. The mass was not mobile and was not attached to the overlying skin. Abdominal CT and pelvic MRI showed a 5 cm tumor in her right internal oblique muscle, with no radiologic evidence of other metastatic disease. MRI showed a 5 cm mass in the oblique muscle that extended to the right ilium (Fig. 2). Biopsy of the mass revealed poorly differentiated carcinoma. 2-Deoxy-2-[^18^F] fluoro-D-glucose position emission tomography demonstrated increased 2-deoxy-2-[^18^F] fluoro-D-glucose uptake by the tumor, with no evidence of other metastatic disease. Laboratory findings were unremarkable. Salvage surgery with partial resection of the oblique muscle and ilium was performed 69 months after the initial operation, and the tumor was completely excised with an adequate surgical margin (Fig. 3). The resulting abdominal wall defect was repaired using dual mesh. Pathological examination of the resected tumor revealed recurrent adenocarcinoma extending from the oblique muscle to the right pelvic wall (Fig. 1A, B). Immunohistochemically, the tumor cells were strongly positive for cytokeratin AE1/AE3 and p53, but negative for estrogen receptor (Fig. 1C). A diagnosis of metastatic cervical cancer (serous carcinoma) was confirmed. She received no further treatment, and is currently free of disease at 10 months after the development of recurrent disease.

### 1.2 Patient consent for publication

Informed written consent was obtained from the patient for publication of this case report and companying images.

## 2 Discussion

We presented our experience with isolated oblique muscle metastasis showing direct invasion of the ilium, which was developed as a first site of recurrence of FIGO stage IB2 cervical adenocarcinoma at 5 years after initial surgery. The patient was successfully treated by extensive surgery, including osteotomy.

The reason for the rarity of skeletal muscle metastasis of cervical cancer remains unclear, although several hypotheses have been postulated:

- contraction of skeletal muscle and constant fluctuations of intramuscular blood pressure act as mechanical barriers to tumor cell implantation;
- local production of lactic acid prevents tumor cell proliferation;
- protease inhibitors in the basement membrane of myocytes inhibit tumor cell invasion; and
- lymphocytes and natural killer cells in skeletal muscles destroy tumor cells.^[2,21,22]^

Cervical cancer is the most common female cancer worldwide. However, as shown in Table 1, our comprehensive literature search found only 19 cases of skeletal muscle metastases developed as sites of recurrence in patients with cervical cancer, indicating the rarity of this condition. The abdominal wall muscles, iliopsoas, and paravertebral muscles are most commonly affected, consistent with previous reports.^[2,3]^ Importantly, of the 19 patients with skeletal muscle metastases, 11 (57.8%) had isolated disease, indicating that skeletal muscle involvement by a recurrent tumor does not necessarily indicate systemic disease.

The risk factors remain largely unknown, although previous reports have suggested that patients with human immunodeficiency virus infection or with advanced cervical cancer are at high risk of skeletal muscle metastasis.^[8,23]^ However, a correlation between such risk factors and skeletal muscle metastasis is not demonstrated in Table 1.

Pain is the most common presenting symptom in patients with skeletal muscle metastasis, and it can be helpful for diagnosis, since soft tissue sarcoma is usually painless, whereas skeletal muscle metastasis frequently presents as a painful soft tissue mass. However, as shown in Table 1, one-third (5/15) of skeletal muscle metastases are asymptomatic. While imaging modalities (contrast-enhanced CT, contrast-enhanced MRI, or 2-deoxy-2-[^18^F] fluoro-D-glucose position emission tomography/CT) are useful for detecting skeletal muscle tumors, they cannot differentiate soft tissue sarcoma from skeletal muscle metastasis.^[4–19]^ Thus, suspected metastasis should be confirmed by fine needle aspiration biopsy.

Although case reports are available, there are no guidelines regarding treatment of this condition due to lack of clinical trials. As shown in Table 1, supportive care alone, chemotherapy, palliative radiotherapy, or chemotherapy following palliative radiotherapy have been tried for multiple metastases. In contrast, surgical excision or definitive radiotherapy with or without adjuvant treatment have been employed for solitary lesions.

The clinical outcome of patients with skeletal muscle metastases in a recurrent setting is generally poor (Table 1). This is at least partly due to the presence of systemic disease, and all 7 patients with no evidence of disease after salvage treatment had isolated skeletal metastasis.^[7,8,10,12,13,17]^ As would be expected, the mortality rate of recurrent cervical cancer patients with isolated skeletal muscle metastasis (18.2%; 2/11) is significantly lower than that of patients with skeletal muscle tumors as part of multiple metastases (75%; 6/8). There may also be other predictors of a favorable outcome. All patients who survived for longer than 1 year after salvage treatment for skeletal muscle metastasis underwent surgery or radiotherapy.^[7,13,17,19]^ Also, there were no deaths from disease progression among the 7 patients with isolated skeletal muscle metastasis who were treated with curative surgery or radiotherapy.^[7,8,10,12,13,17]^ Accordingly, the presence of disseminated tumors at diagnosis of skeletal muscle metastasis and the type of salvage treatment may be predictors of survival for this patient population. Moreover, among the 5 patients with disease-free interval (DFI)>30 months,^[7,8,13,18]^ only 1 patient (20%) died of disease progression and the mortality rate was significantly lower than among patients with DFI < 30 months (7/14, mortality rate of 50%). This suggests that DFI may be a useful prognostic indicator for these patients. We consider that local treatment should be done, either surgery or radiotherapy with or without adjuvant treatment, at least for patients having an isolated tumor with a longer DFI.

In conclusion, we experienced a rare case of isolated oblique muscle metastasis as a first site of recurrence of FIGO stage IB2 cervical adenocarcinoma. Although skeletal muscle metastasis is rare, this condition should be considered during the follow-up period, especially when patients complain of muscular pain with insidious progression. The present case and our literature review highlighted the possibility that loco-regional treatment may be curative in selected recurrent cervical cancer patients with skeletal muscle metastasis. Given the rarity of this condition, we believe it is important to report individual cases so that optimal treatment can be established.

## Author contributions

NO, KI and NK collected data regarding isolated recurrence using the PubMed database.

NO, SM, KI, RK, KH and AN conducted surgical procedure.

SS and HK made substantial contribution to conception of the study.

SS and CO conducted histopathological and immunostaining procedure.

SM contributed to the study design, writing manuscript, and interpretation of included clinical studies.

The final version of the manuscript has been read and approved by all authors.

## Author contributions

**Conceptualization:** Seiji Mabuchi, Hiroshi Kobayashi.

**Data curation:** Nagisa Omokawa, Seiji Mabuchi, Kana Iwai, Naoki Kawahara.

**Funding acquisition:** Hiroshi Kobayashi.

**Investigation:** Seiji Mabuchi, Sumire Sugimoto, Chiho Oobayashi, Kanya Honoki.

**Methodology:** Seiji Mabuchi.

**Resources:** Ryuji Kawaguchi, Kanya Honoki.

**Supervision:** Seiji Mabuchi, Ryuji Kawaguchi.

**Visualization:** Seiji Mabuchi, Sumire Sugimoto, Chiho Oobayashi.

**Writing – original draft:** Seiji Mabuchi.

**Writing – review & editing:** Naoki Kawahara.
